# Correction: Aquatic invasive alien rodents in Western France: Where do we stand today after decades of control?

**DOI:** 10.1371/journal.pone.0303718

**Published:** 2024-05-09

**Authors:** Manon Bonnet, Gérald Guédon, Marc Pondaven, Sandro Bertolino, Damien Padiolleau, Vanessa Pénisson, Francine Gastinel, Fabien Angot, Pierre-Cyril Renaud, Antonin Frémy, Olivier Pays

The title and caption for Fig 7 is missing from the article. The title and caption have been provided here:

**Fig 7:**
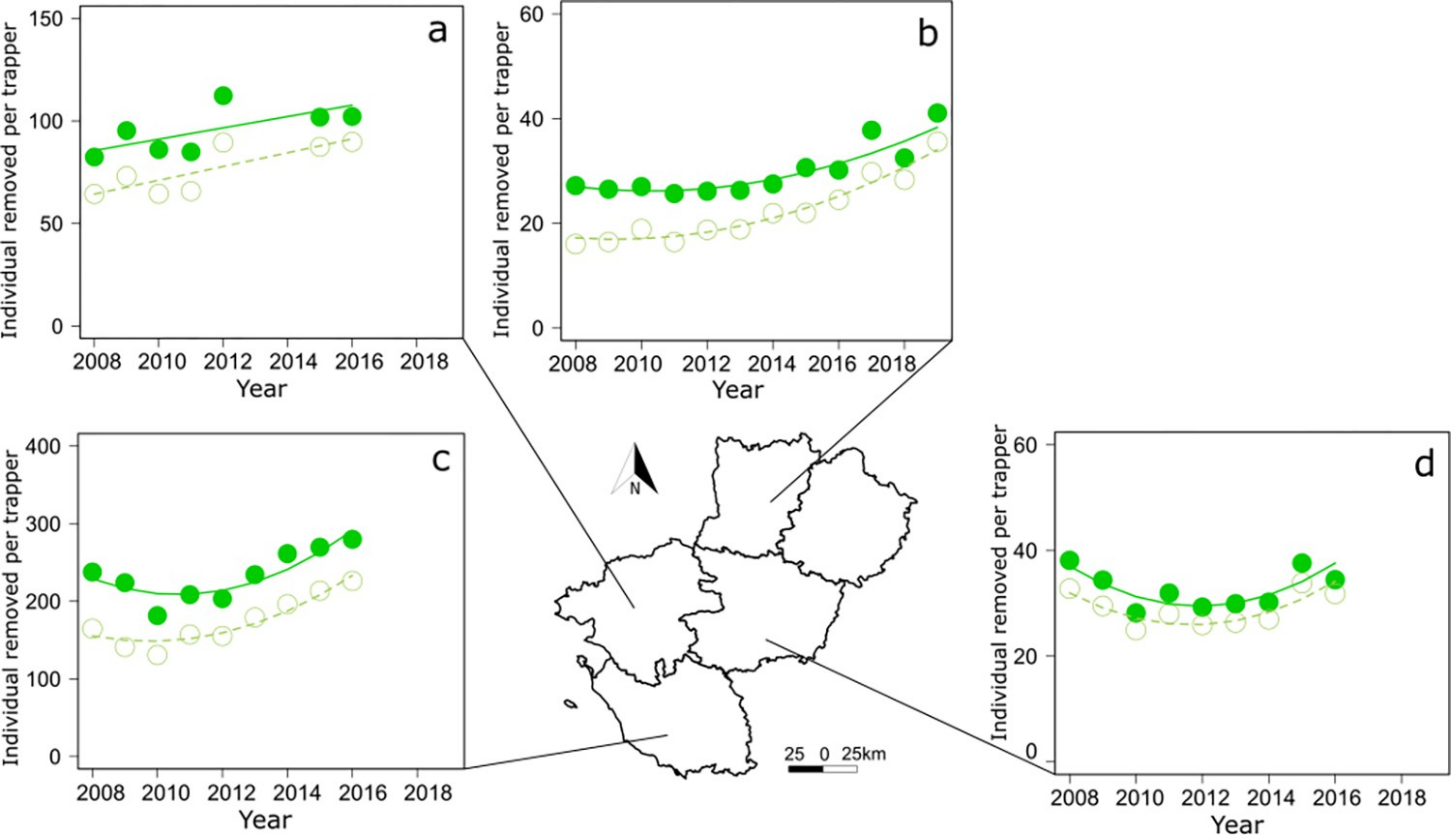
Variation of the number of aquatic invasive alien rodents (AIAR) removed per trapper in the 5 departments of the region Pays de la Loire. AIAR (full dot) and coypus (open dot) removed per trapper in (a) Loire-Atlantique, (b) Mayenne, (c) Vendée, and (d) Maine-et-Loire. Curved lines represent significant trends from generalized least squares (GLS) models with an autoregressive moving average term (see S2 Table for statistical details). Source of the background map: IGN GEOFLA®.
