# Supplementary material for: Aquatic invasive alien rodents in Western France: Where do we stand today after decades of control?
Source: PLoS One. 2021 Apr 8;16(4):e0249904. doi: 10.1371/journal.pone.0249904 (PMC8031452; doi:10.1371/journal.pone.0249904)

**S1 Fig.** **Correlogram of the variation of the Moran’s I index assessed from the number of captures per municipalities.** Open dot indicates that Moran’s I index is not significant. Red full dot indicates a significant Moran’s index with a value being outside the 95% CI assessed from 1000 simulations. This analysis has been run from all data of AIAR captures in 2016 using *pgirmess* package in R. This figure shows that the number of captures in a municipality is indisputably spatially correlated with captures in neighbouring municipalities distant up to 40 km and in a lesser extent up to 90 km.


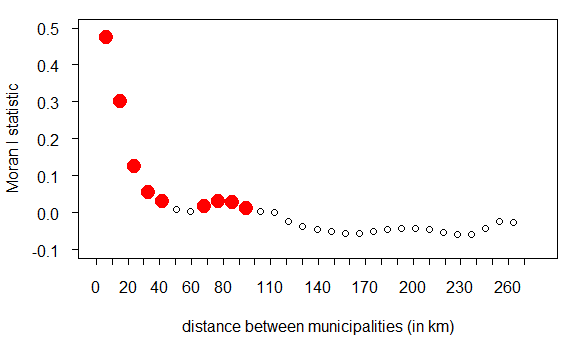

Supplement: S1 Fig — Open dot indicates that Moran’s I index is not significant. Red full dot indicates a significant Moran’s index with a value being outside the 95% CI assessed from 1000 simulations. This analysis has been run from all data of AIAR captures in 2016 using pgirmess package in R. This figure shows that the number of captures in a municipality is indisputably spatially correlated with captures in neighbouring municipalities distant up to 40 km and in a lesser extent up to 90 km. (DOCX) [file pone.0249904.s001.docx]
