# Supplementary material for: Aquatic invasive alien rodents in Western France: Where do we stand today after decades of control?
Source: PLoS One. 2021 Apr 8;16(4):e0249904. doi: 10.1371/journal.pone.0249904 (PMC8031452; doi:10.1371/journal.pone.0249904)
Supplement: S1 Table — (DOCX) [file pone.0249904.s002.docx]

**S1 Table. Data that were available per year and department in the permanent control programme on the Alien Invasive Aquatic Rodents (AIAR) in the region Pays de la Loire.**

| Variables | Year | | | | | | | | | | | |
| --- | --- | --- | --- | --- | --- | --- | --- | --- | --- | --- | --- | --- |
|  | 2008 | 2009 | 2010 | 2011 | 2012 | 2013 | 2014 | 2015 | 2016 | 2017 | 2018 | 2019 |
| **Pays de la Loire** |  |  |  |  |  |  |  |  |  |  |  |  |
| Number of AIAR removed | 186438 | 198695 | 163630 | 187159 | 208315 | 198879 | 251618 | 261989 | 287763 | 238494 | 208259 | 166335 |
| Number of coypus removed (except Sarthe) | 129582 | 129859 | 117293 | 134811 | 151780 | 151500 | 191087 | 208331 | 230990 | - | - | - |
| Number of trappers (except Sarthe) | 1994 | 2125 | 2015 | 2230 | 2321 | - | - | 2652 | 2924 | - | - | - |
|  |  |  |  |  |  |  |  |  |  |  |  |  |
| **Loire-Atlantique** |  |  |  |  |  |  |  |  |  |  |  |  |
| Number of AIAR removed | 42591 | 45536 | 40257 | 42271 | 62200 | 55307 | 79276 | 74934 | 88662 | 63467 | 48671 | 71596 |
| Number of Coypus removed | 33278 | 34926 | 30172 | 32725 | 49551 | 43330 | 65943 | 64291 | 77904 | 57289 | 44384 | 66183 |
| Number of trappers | 517 | 478 | 468 | 498 | 554 | - | - | 736 | 868 | 911 | 759 | 832 |
| Number of municipalities with data on AIAR (percentage of municipalities with data) | 153 (69.23) | 150 (67.87) | 152 (68.78) | 151 (68.33) | 153 (69.23) | 150 (67.87) | 157 (71.04) | 162 (73.30) | 162 (73.30) | 150 (67.87) | 119 (53.85) | - |
|  |  |  |  |  |  |  |  |  |  |  |  |  |
| **Maine-et-Loire** |  |  |  |  |  |  |  |  |  |  |  |  |
| Number of AIAR removed | 26991 | 28068 | 20257 | 25072 | 24849 | 24347 | 27764 | 35438 | 37427 | 32900 | 24505 | - |
| Number of Coypus removed | 23201 | 24080 | 17923 | 21972 | 21997 | 21465 | 24779 | 31860 | 34529 | 30501 | 23161 | - |
| Number of trappers | 708 | 816 | 719 | 785 | 849 | 814 | 919 | 942 | 1086 | - | 831 | - |
| Number of municipalities with data on AIAR (percentage of municipalities with data) | 221 (61.90) | 249 (69.75) | 215 (60.22) | 232 (64.99) | 242 (67.79) | 226 (63.31) | 222 (61.18) | 256 (71.71) | 256 (71.71) | 230 (64.43) | 201 (56.30) | - |
|  |  |  |  |  |  |  |  |  |  |  |  |  |
| **Mayenne** |  |  |  |  |  |  |  |  |  |  |  |  |
| Number of AIAR removed | 9790 | 9860 | 9425 | 12529 | 11908 | 11805 | 12683 | 15317 | 15085 | 18813 | 17404 | 24353 |
| Number of Coypus removed | 5769 | 6111 | 6595 | 8014 | 8546 | 8458 | 10112 | 10987 | 12246 | 14810 | 15172 | 21094 |
| Number of trappers | 360 | 372 | 349 | 488 | 456 | 449 | 461 | 500 | 500 | 498 | 536 | 593 |
| Number of municipalities with data on AIAR (percentage of municipalities with data) | 159 (60.92) | 161 (61.69) | 165 (63.22) | 179 (68.58) | 180 (68.97) | 171 (65.52) | 176 (67.43) | 176 (67.43) | 168 (64.37) | 162 (62.07) | 169 (64.75) | - |
|  |  |  |  |  |  |  |  |  |  |  |  |  |
| **Sarthe** |  |  |  |  |  |  |  |  |  |  |  |  |
| Number of AIAR removed | 9863 | 12460 | 6754 | 11610 | 15405 | 5034 | 11690 | 8501 | 15074 | 18183 | 31625 | 19144 |
| Number of Coypus removed | 8798 | 10488 | 6071 | 10254 | - | - | 9930 | - | - | - | - | 18493 |
| Number of trappers | - | - | - | - | - | - | - | - | - | - | - | 420 |
| Number of municipalities with data on AIAR (percentage of municipalities with data) | - | - | 79 (21.35) | - | - | - | - | 110 (29.73) | 99 (26.76) | 129 (34.86) | 175 (47.30) | - |
|  |  |  |  |  |  |  |  |  |  |  |  |  |
| **Vendée** |  |  |  |  |  |  |  |  |  |  |  |  |
| Number of AIAR removed | 97203 | 102771 | 86937 | 95677 | 93953 | 102386 | 120205 | 127799 | 131515 | 105131 | 86054 | 51242 |
| Number of Coypus removed | 67334 | 64742 | 62603 | 72100 | 71686 | 78247 | 90253 | 101193 | 106311 | 89192 | 77435 | 47887 |
| Number of trappers | 409 | 459 | 479 | 459 | 462 | 437 | 460 | 474 | 470 | - | - | - |
| Number of municipalities with data on AIAR (percentage of municipalities with data) | 69 (24.47) | 86 (30.50) | 84 (29.79) | 173 (61.35) | 167 (59.22) | 166 (58.87) | 247 (87.59) | 248 (87.94) | 242 (85.82) | 241 (85.46) | 267 (94.68) | - |
