# Supplementary material for: Aquatic invasive alien rodents in Western France: Where do we stand today after decades of control?
Source: PLoS One. 2021 Apr 8;16(4):e0249904. doi: 10.1371/journal.pone.0249904 (PMC8031452; doi:10.1371/journal.pone.0249904)
Supplement: S2 Table — Each GLS run is presented with the F-value, the numDF, the p-value and the Pseudo-R2. (DOCX) [file pone.0249904.s003.docx]

**S2 Table**. Statistics from the generalized least squares models with Auto Regressive Moving Average term (ARMA (q=1)) of the effect of time on the number of Alien Invasive Aquatic Rodents (AIAR) removed (coypus and muskrats), coypus only, number of trappers, AIAR removed per trapper and number of municipalities that encountered positive value of inter-annual captures rates of animals (both coypu and muskrat and coypu only). Each GLS run is presented with the F-value, the numDF, the p-value and the Pseudo-R².

| Localisation | Dependant variables | Independant variables | F value | numDF | P value | Pseudo R² |
| --- | --- | --- | --- | --- | --- | --- |
| Pays de la Loire | AIAR removed | (Intercept) | 50,915.14 | 1 | <0.0001 | 89.51 |
|  |  | Time | 331.33 | 1 | <0.0001 |  |
|  |  | Time² | 37.56 | 1 | <0.0001 |  |
|  | Coypu removed | (Intercept) | 58,890.62 | 1 | <0.0001 | 96.59 |
|  |  | Time | 1,068.15 | 1 | <0.0001 |  |
|  |  | Time² | 78.86 | 1 | <0.0001 |  |
|  | Trappers | (Intercept) | 131,876.66 | 1 | <0.0001 | 96.34 |
|  |  | Time | 565.90 | 1 | <0.0001 |  |
|  |  | Time² | 13.51 | 1 | 0.021 |  |
|  | AIAR per trapper | (Intercept) | 799.07 | 1 | <0.0001 | - |
|  |  | Time | 1.50 | 1 | 0.28 |  |
|  |  | Time² | 1.97 | 1 | 0.23 |  |
|  | Coypu per trapper | (Intercept) | 818.66 | 1 | <0.0001 | 68.67 |
|  |  | Time | 9.60 | 1 | 0.027 |  |
|  |  | Time² | 4.87 | 1 | 0.092 |  |
| Loire-Atlantique | AIAR removed | (Intercept) | 1,826.78 | 1 | <0.0001 | 87.46 |
|  |  | Time | 107.47 | 1 | <0.0001 |  |
|  |  | Time² | 5.96 | 1 | 0.050 |  |
|  | Coypu removed | (Intercept) | 3,976.57 | 1 | <0.0001 | 90.08 |
|  |  | Time | 239.79 | 1 | <0.0001 |  |
|  |  | Time² | 15.00 | 1 | 0.0082 |  |
|  | Trappers | (Intercept) | 5,687.12 | 1 | <0.0001 | 99.1 |
|  |  | Time | 298.11 | 1 | <0.0001 |  |
|  |  | Time² | 63.53 | 1 | 0.0013 |  |
|  | AIAR per trapper | (Intercept) | 11,117.52 | 1 | <0.0001 | 28.57 |
|  |  | Time | 17.62 | 1 | 0.0085 |  |
|  |  | Time² | 0.54 | 1 | 0.50 |  |
|  | Coypu per trapper | (Intercept) | 798.49 | 1 | <0.0001 | 60.97 |
|  |  | Time | 11.50 | 1 | 0.019 |  |
|  |  | Time² | 0.13 | 1 | 0.74 |  |
| Maine-et-Loire | AIAR removed | (Intercept) | 31,825.24 | 1 | <0.0001 | 83.15 |
|  |  | Time | 119.86 | 1 | <0.0001 |  |
|  |  | Time² | 66.36 | 1 | <0.0001 |  |
|  | Coypu removed | (Intercept) | 33,770.73 | 1 | <0.0001 | 87.52 |
|  |  | Time | 171.94 | 1 | <0.0001 |  |
|  |  | Time² | 75.57 | 1 | <0.0001 |  |
|  | Trappers | (Intercept) | 81,803.61 | 1 | <0.0001 | 86.53 |
|  |  | Time | 245.62 | 1 | <0.0001 |  |
|  |  | Time² | 16.11 | 1 | 0.007 |  |
|  | AIAR per trapper | (Intercept) | 38,239.45 | 1 | <0.0001 | 57.45 |
|  |  | Time | 0.35 | 1 | 0.57 |  |
|  |  | Time² | 37.93 | 1 | 0.0008 |  |
|  | Coypu per trapper | (Intercept) | 46,079.48 | 1 | <0.0001 | 64.69 |
|  |  | Time | 7.10 | 1 | 0.037 |  |
|  |  | Time² | 47.70 | 1 | 0.0005 |  |
| Mayenne | AIAR removed | (Intercept) | 7,324.06 | 1 | <0.0001 | 90.34 |
|  |  | Time | 335.20 | 1 | <0.0001 |  |
|  |  | Time² | 21.41 | 1 | 0.0012 |  |
|  | Coypu removed | (Intercept) | 1,407.05 | 1 | <0.0001 | 95.65 |
|  |  | Time | 204.68 | 1 | <0.0001 |  |
|  |  | Time² | 18.73 | 1 | 0.0019 |  |
|  | Trappers | (Intercept) | 2,435.67 | 1 | <0.0001 | 81.15 |
|  |  | Time | 46.62 | 1 | <0.0001 |  |
|  |  | Time² | 0.0001 | 1 | 0.99 |  |
|  | AIAR per trapper | (Intercept) | 109,984.61 | 1 | <0.0001 | 84.27 |
|  |  | Time | 440.81 | 1 | <0.0001 |  |
|  |  | Time² | 68.95 | 1 | <0.0001 |  |
|  | Coypu per trapper | (Intercept) | 112,726.01 | 1 | <0.0001 | 94.52 |
|  |  | Time | 1,546.63 | 1 | <0.0001 |  |
|  |  | Time² | 94.46 | 1 | <0.0001 |  |
| Sarthe | AIAR removed | (Intercept) | 161.23 | 1 | <0.0001 | 50.85 |
|  |  | Time | 15.19 | 1 | <0.0001 |  |
|  |  | Time² | 6.23 | 1 | 0.034 |  |
| Vendée | AIAR removed | (Intercept) | 2,237.79 | 1 | <0.0001 | 86.89 |
|  |  | Time | 30.34 | 1 | 0.0015 |  |
|  |  | Time² | 9.90 | 1 | 0.020 |  |
|  | Coypu removed | (Intercept) | 132,124.44 | 1 | <0.0001 | 96.84 |
|  |  | Time | 1,109.89 | 1 | <0.0001 |  |
|  |  | Time² | 76.29 | 1 | <0.0001 |  |
|  | Trappers | (Intercept) | 2,049.12 | 1 | <0.0001 | - |
|  |  | Time | 1.91 | 1 | 0.21 |  |
|  |  | Time² | 0.75 | 1 | 0.42 |  |
|  | AIAR per trapper | (Intercept) | 1,010.56 | 1 | <0.0001 | 84.27 |
|  |  | Time | 8.22 | 1 | 0.029 |  |
|  |  | Time² | 6.46 | 1 | 0.044 |  |
|  | Coypu per trapper | (Intercept) | 1,167.47 | 1 | <0.0001 | 90.17 |
|  |  | Time | 26.95 | 1 | 0.0020 |  |
|  |  | Time² | 7.93 | 1 | 0.031 |  |
